# The effects of the surface-exposed residues on the binding and hydrolytic activities of *Vibrio carchariae *chitinase A

**DOI:** 10.1186/1471-2091-9-2

**Published:** 2008-01-21

**Authors:** Supansa Pantoom, Chomphunuch Songsiriritthigul, Wipa Suginta

**Affiliations:** 1 School of Biochemistry, Suranaree University of Technology, Nakhon Ratchasima 30000, Thailand; 2 National Synchrotron Research Center, Nakhon Ratchasima 30000, Thailand

## Abstract

**Background:**

*Vibrio carchariae *chitinase A (EC3.2.1.14) is a family-18 glycosyl hydrolase and comprises three distinct structural domains: i) the amino terminal chitin binding domain (ChBD); ii) the (α/β)_8 _TIM barrel catalytic domain (CatD); and iii) the α + β insertion domain. The predicted tertiary structure of *V. carchariae *chitinase A has located the residues Ser33 & Trp70 at the end of ChBD and Trp231 & Tyr245 at the exterior of the catalytic cleft. These residues are surface-exposed and presumably play an important role in chitin hydrolysis.

**Results:**

Point mutations of the target residues of *V. carchariae *chitinase A were generated by site-directed mutagenesis. With respect to their binding activity towards crystalline *α*-chitin and colloidal chitin, chitin binding assays demonstrated a considerable decrease for mutants W70A and Y245W, and a notable increase for S33W and W231A. When the specific hydrolyzing activity was determined, mutant W231A displayed reduced hydrolytic activity, whilst Y245W showed enhanced activity. This suggested that an alteration in the hydrolytic activity was not correlated with a change in the ability of the enzyme to bind to chitin polymer. A mutation of Trp70 to Ala caused the most severe loss in both the binding and hydrolytic activities, which suggested that it is essential for crystalline chitin binding and hydrolysis. Mutations varied neither the specific hydrolyzing activity against *p*NP-[GlcNAc]_2_, nor the catalytic efficiency against chitohexaose, implying that the mutated residues are not important in oligosaccharide hydrolysis.

**Conclusion:**

Our data provide direct evidence that the binding as well as hydrolytic activities of *V. carchariae *chitinase A to insoluble chitin are greatly influenced by Trp70 and less influenced by Ser33. Though Trp231 and Tyr245 are involved in chitin hydrolysis, they do not play a major role in the binding process of crystalline chitin and the guidance of the chitin chain into the substrate binding cleft of the enzyme.

## Background

Chitin is a homopolysaccharide chain of *N*-acetylglucosamine (GlcNAc or G1) units combined together with *β*-1,4 glycosidic linkages. Chitin is one of the most abundant biopolymers found in nature as constituent of fungal cell walls and exoskeletons of crustaceans and insects. However, the *β*-GlcNAc units that generally form intra- and intermolecular H-bonds make chitin completely insoluble in water and its use is thus limited. Several strategies have been developed for converting chitin into small soluble derivatives, which are more useful for applications in the fields of medicine, agriculture and industry. Enzymatic degradation of chitin using biocatalysts seems to be the method of choice since the type, quantity and quality of oligomeric products can be well controlled and the reaction occurs quickly and completely under mild conditions without generation of environmental pollutants.

**Figure 1:**
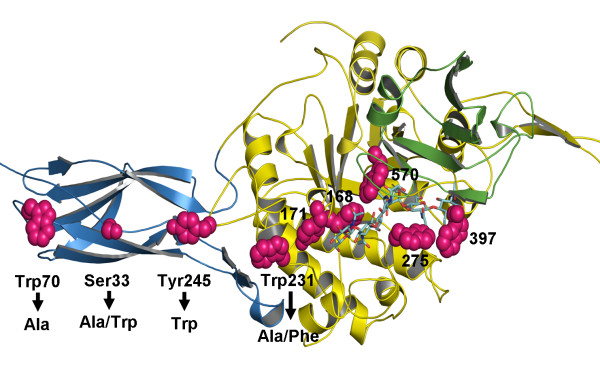
**The Swiss-Model 3D-structure of *V. carchariae *chitinase A**. A ribbon representation of the 3D-structure of *V. carchariae *chitinase A was constructed based on the x-ray structure of *S. marcescens *Chi A E315L mutant as described in the text. The *N*-terminal chitin binding domain is presented in cyan, the TIM barrel domain in yellow and the small insertion domain in green. The coordinates of [GlcNAc]_6 _that are modeled in the active site of the *Vibrio *enzyme are shown as a stick model with N atoms in blue and O atoms in red. The mutated residues (Ser33, Trp70, Trp231 and Tyr245) and other substrate binding residues are also presented in stick model (magenta).

Chitinases (EC3.2.1.14) are a diverse group of enzymes that catalyze the conversion of insoluble chitin to soluble oligosaccharides. They are found in a wide variety of organisms including virus, bacteria, fungi, insects, plants and animals [1-8]. In the carbohydrate active enzymes (CAZy) database , carbohydrate enzymes are first classified as glycosyl hydrolases (GH), glycosyl transferases (GT), polysaccharide lyases (PL), carbohydrate esterases (CE), and carbohydrate binding modules (CBM), and then further divided into numbered families with structurally-related catalytic and carbohydrate-binding modules. Following this classification, chitinases are commonly listed as family GH-18 and family GH-19 enzymes. Family-18 chitinases have the catalytic crevice located at top of the (α/β)_8_-TIM barrel domain [9,10], whereas the catalytic domain of family-19 chitinases comprises two lobes, each of which is rich in α-helical structure [11]. Bacteria such as *Serratia marcescens*, *Bacillus circulans*, *Alteromonas sp*. and marine *Vibrios *produce chitinases to synergistically degrade chitin and use it as a sole source of energy [1,12-16]. The mechanism of chitin degradation by bacterial chitinases was mainly derived from the outcome of structural studies or site-directed mutagenesis [17-20]. Unlike chitooligosaccharides, chitin polymer has been presumed to unidirectionally enter the substrate binding cleft of chitinases under the guidance of a few surface-exposed residues at the exterior of the substrate binding cleft [21,22]. Those residues were identified as Trp33, Trp69, Phe232, and Trp245 in *S. marcescens *Chi A [18], Ser33, Trp70, Trp232, and Trp245 in *Aeromonas caviae *Chi1 [23], and Trp122 and Trp134 in *B. circulans *Chi A1 [17]. Structurally, the latter two residues are located in the equivalent locations of Tyr245 and Trp231, respectively of *V. carchariae *chitinase A (Fig. 1 and ref [17]). We previously isolated chitinase A from a marine bacterium, *Vibrio carchariae *[24]. The enzyme was found to be highly expressed upon induction with chitin and was active as a monomer of 63 kDa. The DNA fragment encoding the functional chitinase A was subsequently cloned into the pQE60 expression vector that was compatible to be highly expressed in *E. coli *type strain M15 [25]. Mutational studies confirmed that the conserved Glu315 acts as the catalytic residue in the substrate-assisted mechanism [26,27], whereas the aromatic residues including Trp168, Tyr171, Trp275, Trp397 and Trp570 participated in direct interactions with chitooligosaccharides [28]. In this study, site-directed mutagenesis was employed in combination with following chitin binding assays and kinetic analysis to investigate the significance of the putative surface-exposed residues Ser33, Trp70, Trp231 and Tyr245 for the binding and hydrolytic activities of the *Vibrio *chitinase A.

## Results

### Homology modeling and sequence analysis

We previously reported gene isolation and sequence analysis of *V. carchariae *chitinase A precursor [25]. Site-directed mutation of the active site residues showed that Glu315 plays an essential role in catalysis [26]. On the other hand, the conserved aromatic residues (Trp168, Tyr171, Trp275, Trp397 and Trp570) located within the substrate binding cleft of the enzyme were found to be important in binding to chitooligosaccharides [28]. Here, we have investigated the functional roles of four amino acid residues (Ser33, Trp70, Trp231 and Tyr245) at the surface of *V. carchariae *chitinase A. All of these residues have been proposed as functionally relevant to binding and hydrolysis of crystalline chitin [17,18]. Fig. 1 represents the modeled 3D-structure of *V. carchariae *chitinase A that was built based upon the crystal structure of *S. marcescens *chitinase A mutant E315L complexed with a chitohexamer [see Methods]. It can clearly be seen that the four residues linearly align with each other. Trp70 and Ser33 are positioned at the end of the *N*-terminal chitin binding domain (ChBD), whilst Trp231 and Tyr245 are found outside the substrate binding cleft where they are part of the TIM barrel catalytic domain.

When the amino acid sequences of several bacterial chitinases were compared, the *V. carchariae *chitinase A (Q9AMP1) exhibited highest sequence identity with chitinase A from *V. harveyi *HY01 (A6AUU6) (93%), moderate identity with chitinase A from *S. marcescens *(P07254) and *Enteromonas sp*. (Q4PZF3) (47%), and low identity with chitinase A1 from *B. circulans *(22%). Surprisingly, extremely low sequence identity was observed when *V. carchariae *chitinase A was compared with chitinase A from *V. splendidus *(A3UMC6) (13%) and *V. cholerae *(A6ACY6) (11%).

**Figure 2:**
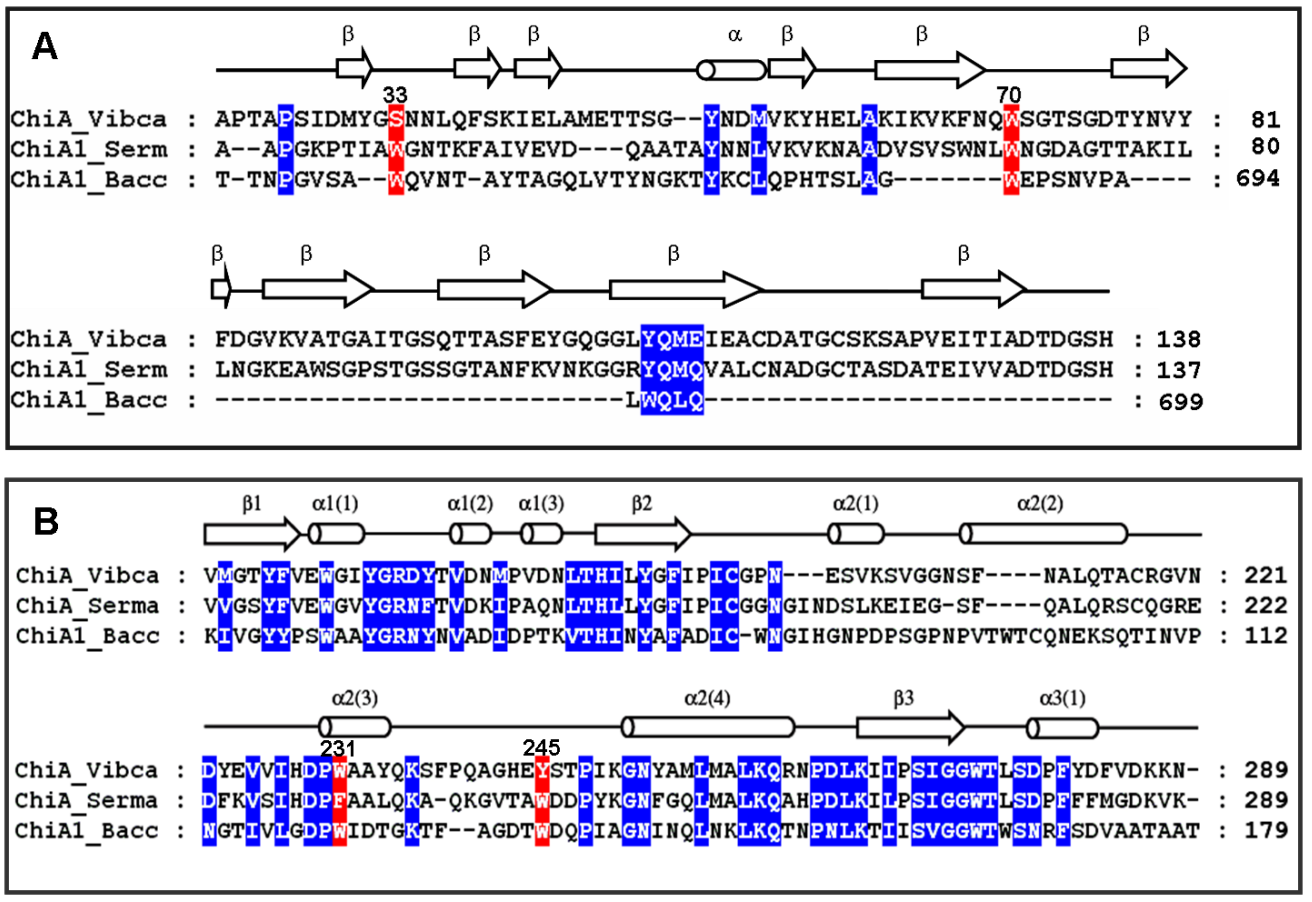
**A structure-based alignment of *V. carchairae *chitinase A with *S. marcescens *chitinase A and *B. circulans *chitinase A1**. A) The *N*-terminal ChBDs of *V. carchariae *chitinase A (residues 22–138) and *S. marcescens *chitinase A (residues 24–137) were aligned with the *C*-terminal fragment (residues 648–699), covering the ChBD of *B. circulans *WL-12 chitinase A1. B) An alignment of the catalytic domain of the three bacterial chitinases with residues 160 to 289 of *V. carchariae *chitinase A being displayed. The chitinase sequences were retrieved from the Swiss-Prot/TreEMBL protein databases, aligned using "MegAlign" in the DNASTAR package, and displayed in Genedoc. The secondary structure of *V. carchariae *chitinase A was predicted from the PHD method in PredictProtein using *S. marcescens *as template [see texts]. Conserved residues are shaded in blue, whereas the residues that are aligned with Ser33, Trp70, Trp231, and Tyr245 of *V. carchariae *chitinase A are shaded in red. ChiA_Vibca: *V. carchariae *chitinase A (Q9AMP1), ChiA_Serma: *S. marcescens *chitinase A (P07254), and ChiA1_Bacc: *B. circulans *chitinase A1 (P20533). *β*-strand is represented by an arrow, *α*-helix by a cylinder and loop by a straight line.

A structure-based alignment of three chitinases, including *V. carchariae *chitinase A, *S. marcescens *chitinase A, and *B. circulans *WL-12 chitinase A1 was constructed and is displayed in Fig. 2A &2B. Fig. 2A represents an alignment of the *N*-terminal ChBDs of the *Vibrio *and *Serratia *chitinases with the *C*-terminal fragment that covers the ChBD of the *Bacillus *chitinase A1 (ChBD_chiA1_). The ChBD_chiA1_consists of the residues 655 to 699 and deletion of this domain led to a severe loss in the binding activity to chitin as well as in the colloidal chitin-hydrolyzing activity, suggesting that this domain is essential for binding to insoluble chitin of this enzyme [29]. As shown in Fig. 2A, the residues Trp656 and Trp687 of the ChBD_chiA1 _are well aligned with Ser33 and Trp70 of *V. carchariae *chitinase A. However, the determination of the solution structure of the ChBD_chiA1 _by Ikegami et al. [30] identified only Trp687 as a putative chitin binding residue, in addition to His681, Thr682, Pro689, and Pro693.

With respect to the alignment of the catalytic domain (Fig. 2B), Trp231 of the *Vibrio *chitinase is equivalent to Trp122 and to Phe232 of the *Bacillus *and *Serratia *chitinases. For Tyr245, this residue is replaced by Trp134 and Trp245 in the *B. circulans *and *S. marcescens *sequences, respectively. The sequence alignment additionally demonstrates the residues Ser33 and Trp70 within the flexible loops that join two strands in the chitin binding region. The residues Trp245 and Tyr231 are found as part of the catalytic (α/β)_8 _TIM barrel, where Tyr245 is exposed on the loop that joins helices 2(3) and 2(4) together and Trp231 is the only residue being found in an α-helix (helix 2(3)).

### Expression and purification of chitinase A and mutants

To investigate the binding and hydrolytic activities of *V. carchariae *chitinase A, target residues as named above were mutated by sited-directed mutagenesis. According to the employed system, the recombinant chitinases were expressed as the *C*-terminally (His)_6 _tagged fusion protein (see Methods). After single-step purification using Ni-NTA agarose affinity chromatography, the yields of the purified proteins was estimated to be approx. 20 to 25 mg/ml per litre of bacterial culture. As analyzed by SDS-PAGE, all the mutated proteins displayed a single band of molecular weight of 63 kDa (data not shown), which is identical to the molecular weight of the wild-type enzyme.

### Effects of mutations on the chitin binding activities of chitinase A

**Figure 3:**
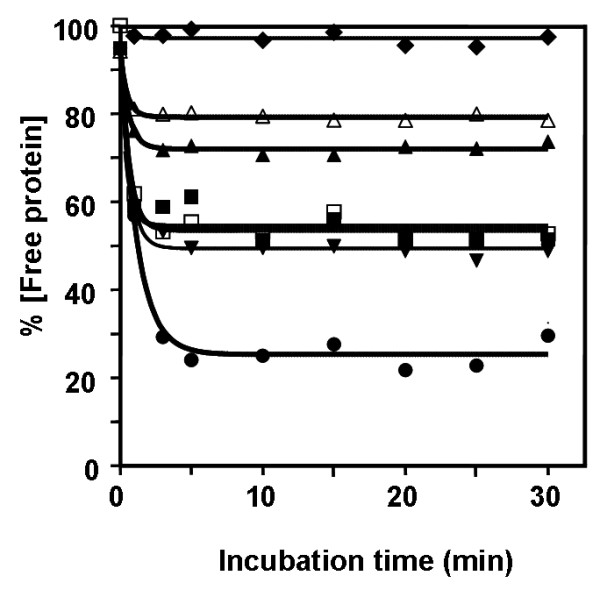
**Time-course of binding of chitinase A and mutant enzymes to colloidal chitin.** Chitinases (1 μmol in 100 mM sodium acetate buffer, pH 5.5) were incubated with 1.0 mg colloidal chitin at 0°C. Decreases in free enzyme concentration were determined at different time points from 0–30 min by Bradford's method. Each data value was calculated from triplicate experiments. Symbols: wild-type (black square); S33A (black upward-pointing triangle); S33W (black downward-pointing triangle); W70A (black diamond); W231A (black circle); W231F (open square); and Y245W (open triangle).

To minimize hydrolysis, all the binding experiments were carried out on ice. Bindings of the wild-type chitinase and mutants to colloidal chitin were initially investigated as a function of time. After a removal of the enzyme bound to chitin, decreases in concentration of the unbound enzyme remaining in the supernatant was monitored discretely at different time points of 0 to 120 min. Fig. 3 demonstrates that the binding process took place rapidly and reached equilibrium within 5 min. The relative binding activity of each mutant to colloidal chitin is following the order W231A > S33W > WT ≅ W231F > S33A > Y245W > W70A.

**Figure 4:**
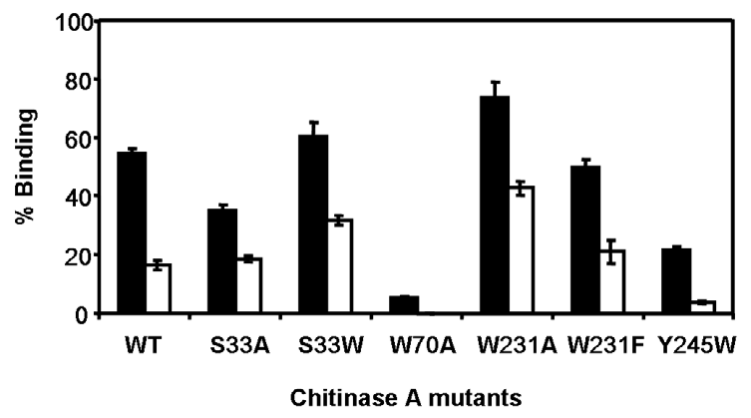
**Binding of chitinase A and mutants to insoluble chitin**. The binding assay set as described in the text was incubated with crystalline *α*-chitin or colloidal chitin for 60 min. The % binding = $\left[\frac{{\text{E}}_{\text{t}}{\text{-E}}_{\text{f}}}{{\text{E}}_{\text{t}}}\right]\times \text{100}$; where E_t _is initial enzyme concentration and E_f _is the free enzyme concentration after binding. Closed and open bars represent % binding to colloidal chitin and crystalline *α*-chitin, respectively. The presented data are mean values obtained from three independent sets of the experiment.

The binding activity of the individual mutants relative to the one of the wild-type enzyme was further examined with colloidal chitin and *α*-chitin polysaccharides at a single time point of 60 minutes. In general, the wild-type and mutant chitinases expressed greater binding activity towards colloidal chitin. A comparison of the level of binding of the engineered enzymes to the two substrates revealed a similar pattern (Fig. 4). For both polysaccharides, W70A and Y245W displayed lower binding activity than the wild-type enzyme. S33A and W231F showed a modest increase in binding to crystalline *α*-chitin and decreased level of binding to colloidal chitin. Mutants S33W and W231A, on the other hand, displayed higher effectiveness in the binding to both substrates. Of all, mutant W231A displayed highest binding activity and W70A exhibited lowest activity. Especially, no detectable binding to crystalline *α*-chitin was observed with mutant W70A.

**Figure 5:**
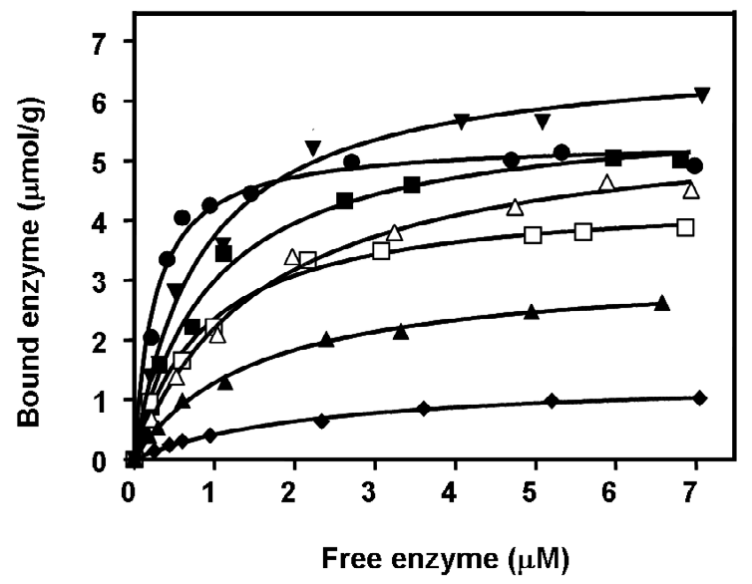
**Equilibrium adsorption isotherms of wild-type and mutant chitinases A to colloidal chitin**. The reaction assay (500 μl) contained 1.0 mg chitin and varied concentrations of enzyme from 0 to 7.0 μM. After 60 min of incubation at 0°C, the reaction mixture was centrifuged and concentrations of E_b _and E_f _were determined as described in the text. Symbols: wild-type (black square); S33A (black upward-pointing triangle); S33W (black downward-pointing triangle); W70A (black diamond); W231A (black circle); W231F (open square); and Y245W (open triangle).

Adsorption isotherms of chitinase mutants to colloidal chitin were carried out relative to that of the wild-type enzyme. Fig. 5 represents a non linear plot of the adsorption isotherms obtained at a fixed concentration of colloidal chitin but varied concentrations of the enzyme (See Methods). In comparison to the wild-type enzyme, mutants S33W and W231A exhibited significantly higher binding activity, whereas mutants W70A, S33A, W231F and Y245W had a notably decreased binding activity. When the dissociation binding constants (*K*_d_) were estimated from the non-linear regression function, it was found that the *K*_d _value of wild-type (0.95 ± 0.11 μM) was slightly larger than the *K*_d _values of S33W (0.84 ± 0.09 μM) and W231F (0.88 ± 0.09 μM), but remarkably greater than the value of W231A (0.26 ± 0.03 μM). In contrast, significantly higher *K*_d _values than the wild-type value were observed with S33A (1.50 ± 0.11 μM), W70A (2.30 ± 0.25 μM), and Y245W (1.60 ± 0.16 μM). These estimated *K*_d _values gave a notation of the enzyme's binding strength in the following order W231A > S33W > W231F > wild-type > S33A > Y245W > W70A, which is in absolute accordance with the binding activities determined by the chitin binding assay (see Fig. 4) and the kinetic data as described below.

### Effects of mutations on the hydrolytic activities of chitinase A

The effects of mutations on the hydrolytic activity of *V. carchariae *chitinase A were further studied by exposing the wild-type and modified enzymes to *p*NP-[GlcNAc]_2_, colloidal chitin and crystalline *α*-chitin. The specific hydrolyzing activity for the three different substrates was subsequently determined. From all the mutants, only W231A displayed a slightly reduced specific hydrolyzing activity against the *p*NP substrate (Table 1).

**Table 1:** Specific hydrolyzing activity of chitinase A and mutants. The reducing sugar assay was carried out against crystalline and colloidal chitin. The release of the hydrolytic products was calculated from a standard curve of [GlcNAc]_2_. On the other hand, the specific hydrolyzing activity against *p*NP-[GlcNAc]_2 _was determined by the colorimetric assay. The release of *p*NP was estimated from a standard curve of *p*NP

**Protein**	**Specific hydrolyzing activity (U/μmol protein)^a^**		
	**Crystalline chitin**	**Colloidal chitin**	***p*NP-[GlcNAc]_2_**
Wild-type	0.59 ± 0.02(100)^b^	12.9 ± 0.22 (100)	50.5 ± 1.13 (100)
S33A	n.d.^c^	8.5 ± 0.50 (66)	58.0 ± 1.08 (115)
S33W	1.00 ± 0.08 (166)	15.3 ± 0.32 (119)	54.0 ± 2.55 (107)
W70A	n.d.	4.3 ± 0.17 (33)	52.8 ± 2.14 (105)
W231A	n.d.	6.6 ± 0.26 (51)	45.2 ± 2.00 (90)
W231F	n.d.	9.2 ± 0.49 (71)	54.3 ± 2.85 (108)
Y245W	1.49 ± 0.09 (250)	19.6 ± 0.53 (152)	53.6 ± 1.61 (106)

^a ^One unit of chitinase is defined as the amount of enzyme that releases 1 μmol of [GlcNAc]_2 _or 1 nmol of *p*NP per min at 37°C.

^b ^Values in parentheses represent relative specific hydrolyzing activities (%).

^c ^Non-detectable activity.

Unlike the *p*NP-glycoside, strong effects on the hydrolytic activities were observed with the insoluble polymeric substrates. The hydrolyzing activity against crystalline *α*-chitin was completely abolished in case of mutants S33A, W70A and W231A/F but improved for mutants S33W and Y245W at levels of 166% and 250% of the wild-type activity, respectively. A similar trend was also seen with colloidal chitin. However, for this substrate S33A, W70A and W231A/F not completely abolished but markedly decreased hydrolyzing activity, while S33W (119%) and Y245W (152%) again where the ones that displayed higher activity than wild-type enzyme. The most severe loss of the specific hydrolyzing activity towards colloidal chitin was detected for mutant W70A, which had a substitution of Trp70 to Ala.

### Steady-state kinetics of chitinase A and mutants

The kinetic parameters of the hydrolytic activity of chitinase A and mutants were finally determined with chitohexaose and colloidal chitin as substrates. As presented in Table 2, mutations of the chosen residues led for the response of enzyme values towards the hexachitooligomer to concomitant decreases in both the *K*_m _and *k*_cat _values. For all the mutants, however, the overall catalytic efficiency (*k*_cat_/*K*_m_) was not much different to the value observed for the wild-type enzyme. In contrast, the kinetic properties of the enzyme against colloidal chitin were significantly modified by the mutations. The *K*_m _values of S33A (2.07 10^1^ mg ml^-1^), W70A (2.26 10^1^ mg ml^-1^), and Y245W (1.82 10^1^ mg ml^-1^) were higher than the wild-type *K*_m _(1.74 10^1^ mg ml^-1^) whereas S33W (1.58 10^1^ mg ml^-1^) and W231A (1.01 10^1^ mg ml^-1^) had considerably lower *K*_m _compared to the reference value. Mutations that caused a large decrease in the enzyme's catalytic activity, *k*_cat_, were Ser33 to Ala, Trp70 to Ala and Trp231 to Ala/Phe, whereas mutations of Ser33 to Trp and Tyr245 to Trp elevated *k*_cat _values instead. The overall catalytic efficiency (*k*_cat_/*K*_m_) that was calculated for the hydrolysis of colloidal chitin was to more or less extent either reduced (for S33A, W70A, W231A and W231F) or increased (for S33W and Y245W).

**Table 2:** Kinetic parameters of substrate hydrolysis by chitinase A wild-type and mutants. A kinetic study was carried out using 0–5% (w/v) colloidal chitin and 0–500 μM chitohexaose as substrates. After 10 minutes of incubation at 37°C, the amounts of the reaction products were determined from a standard curve of [GlcNAc]_2_

**Chitinase A variant**	**Chitohexaose**			**Colloidal chitin**		
	***K*_m_(μM)**	***k*_cat _(s^-1^)**	***k*_cat_/*K*_m_(s^-1^M^-1^)**	***K*_m_(10^1^ mg ml^-1^)**	***k*_cat _(s^-1^)**	***k_cat_*/*K*_m_(10^-1^ s^-1^/mg ml^-1^)**
Wild-type	218 ± 22.0	2.9	1.3 × 10^3 ^(100)^a^	1.74 ± 0.1	1.2	0.7 (100)
S33A	171 ± 20.5	2.6	1.5 × 10^3 ^(115)	2.07 ± 0.2	0.9	0.4 (57)
S33W	210 ± 15.4	2.8	1.3 × 10^3 ^(100)	1.58 ± 0.2	1.4	0.9 (129)
W70A	185 ± 22.0	2.5	1.3 × 10^3 ^(100)	2.26 ± 0.4	0.4	0.2 (29)
W231A	189 ± 13.2	2.6	1.4 × 10^3 ^(108)	1.01 ± 0.2	0.6	0.6 (86)
W231F	163 ± 10.3	2.4	1.5 × 10^3 ^(115)	1.70 ± 0.2	0.9	0.5 (71)
Y245W	201 ± 13.1	2.6	1.3 × 10^3 ^(100)	1.82 ± 0.2	1.7	0.9 (129)

^a ^Relative catalytic efficiencies (%) are shown in parentheses.

## Discussion

This study describes the possible role of Ser33, Trp70, Trp231, and Tyr245 in chitin binding and hydrolysis. Point mutations of these residues were introduced by site-directed mutagenesis and changes in the binding and hydrolytic activities of the enzyme as a result of the amino acid substitution were subsequently investigated for various substrates. Chitin binding assays (Fig. 3 &4) demonstrate a decrease in the binding activity of mutants S33A, W70A and Y245W to various extents. However, the most severe effect was observed with W70A. A time course study displayed no binding activity of W70A but retained activity of other mutants to colloidal chitin (Fig. 3). This strongly suggested that Trp70 is the major determinant for insoluble chitin binding. Sugiyama and colleagues previously employed the reducing-end labeling technique and the tilt micro-diffraction method [21,22] to illustrate the molecular directionality of crystalline *β*-chitin hydrolysis by *S. marescens *chitinase A and *B. chitinase *chitinase A1. If the chitin polymer enters the substrate binding cleft of *V. carchariae *chitinase A from the reducing end as described for the *Serratia *and *Bacillus *enzymes, Trp70 will likely serve as a platform for the arrival of a chitin molecule. This idea is well complimented by the location of the residue at the end of ChBD. A remarkable loss of the specific hydrolyzing activity as well as the decrease in the rate of enzyme turnover (*k*_cat_) that was observed for mutant W70A is hence explainable by the loss of the binding strength due to a substitution of Trp to Ala and an associated change in the hydrophobic interactions. A similar performance was seen for residue Ser33. Although to a lesser extent, a mutation of Ser33 to Ala also led to decreased binding activity, while its mutation to Trp improved binding activity. This finding provides additional evidence that binding of the chitin chain to the ChBD is cooperatively taken place via hydrophobic interactions and influenced by the molecular setting in this region.

The most striking observations were made with the residue Trp231. As demonstrated by the modeled 3D-structure (see Fig. 1), the residue Trp231 is placed at the outermost of the catalytic surface, thereby lying closest to the non-reducing end of the substrate binding cleft. The observed drastic improvement (rather than reduction) in the binding efficiency of mutant W231A could only be explained as a removal of the side-chain blockage. Hence, the reduced hydrolyzing activity of mutant W231A was unlikely influenced by changes in the binding acitivity as a result of the alanine substitution of Trp231. Apparently, the same phenomenon was previously recognized in *S. marcescens *chitinase A [18], with which a mutation of Phe232 to Ala seriously diminished the hydroyzing acitivty but left the binding activities to both colloidal chitin and *β*-chitin microfibrils unchanged.

When the next residue in line (Tyr245) (see Fig. 1) was mutated to a bulkier side-chain (Trp), inverse effects (reduced binding but improved hydrolysis) were observed. This complimented the idea of the binding barrier around the entrance hall of the catalytic domain by Trp231 and Tyr245. Similar findings were also recognized with a cellulose degrading enzyme, *Thermobifida fusca *endoglucanase (Cel9A) [31]. With this enzyme, it was observed that mutations of the surface-exposed cellulose binding residues Arg557 and Glu559 to Ala (mutant R557A/E559A) led to a severe loss in the hydrolytic activity against crystalline cellulose, but a change in the binding activity was not at all observed. Structurally, the residues Arg557 and Glu559 are found on the surface of the cellulose binding module (CBM), closest to the catalytic binding cleft of Cel9A. Therefore, the effects of Arg557 and Glu559 would be explained in analogy to those of Trp231 and Tyr245 in *V. carchariae *chitinase A.

In marked contrast, observations made with the *Vibrio *Trp231 mutation were different to the studies of Li et al. on *A. caviae *Chi 1 [23] and of Watanabe et al. on *B. circulans *Chi A1 [17]. Mutations of Trp232 and Trp245 (in *A. caviae *Chi 1) or Trp122 and Trp 134 (in *B. circulans*) to alanine resulted in a marked loss in both binding and hydrolyzing acitivities, especially againts crystalline *β*-chitin. Therefore, the reduced hydrolytic activities were assumed to be associated with the weaker binding of the two corresponding residues. Based on their mutational data, the residues seemed to participate directly in binding to crystalline chitin, and subsequently cooperatively assisting the chitin chain to penetrate through the catalytic cleft of *A. caviae *or *B. circulans *chitinase.

Indeed, the above-mentioned event that took place in the *A. carviae *and *B. circulans *chitinases did not seem to be the case for the *V. carchariae *chitinase A due to different behaviors of Trp231 and Tyr245 found for the *Vibrio *enzyme. Our data suggested that a possible action of *V. carchariae *chitinase A on insoluble chitin could proceed as follows: i) Initial binding of a chitin chain to the ChBD. This process is most influenced by the hydrophobic interaction set between the incoming sugar and residue Trp70, which is located at the doorway of the ChBD; ii) Further binding of GlcNAc units. However, binding through Ser33 remains inconclusive, since the mutational results revealed that Ser33 did not act as a powerful binding residue. Alternatively, this binding step might be made through a different surface-exposed aromatic residue located nearby; and iii) Sliding of bound sugar units of the chitin chain into the substrate binding cleft. Based on the 'slide and bend' mechanism proposed by Watanabe and others [17,21,32], the sliding process is achieved by cooperative interactions with other surface-exposed aromatic residues located close to the entrance of the substrate binding cleft. However, our data strongly suggested that the chitin chain movement most likely takes place via an interaction with different surface-exposed aromatic residues other than Tyr245 and Trp231.

When *p*NP-[GlcNAc]_2 _was used as a substrate, hydrolyzing activities of the mutated enzymes and the wild-type enzyme were almost indistinguishable. This observation and the essentially unchanged catalytic efficiency (k_cat_/*K*_m_) of all mutants compared to wild-type enzyme clearly pointed out that Ser33, Trp70, Trp231 and Tyr245 do not play a major role in the process of hydrolysis of soluble chitooligosaccharides.

## Conclusion

Point mutations of four surface-exposed residues of *V. carchariae *chitinase A and subsequent experiments on chitin binding and hydrolysis were performed. Trp70, which is located at the *N*-terminal end of the chitin binding domain, was identified as the most crucial residue in colloidal and crystalline chitin binding and consequently their hydrolysis. The residues Trp231 and Tyr245, both located nearer to the substrate-binding cleft, influenced chitin hydrolysis but not really insoluble chitin binding.

## Methods

### Bacterial strains and expression plasmid

*Escherichia coli *type strain DH5α was used for routine cloning, subcloning and plasmid preparation. Supercompetent *E. coli *XL1Blue (Stratagene, La Jolla, CA, USA) was the host strain for the production of mutagenized DNA.*E. coli *type strain M15 (Qiagen, Valencia, CA, USA) and the pQE 60 expression vector harboring *chitinase A *gene fragments were used for a high-level expression of recombinant chitinases.

### A structural based sequence alignment and homology modeling

The amino acid sequence alignment was constructed by the program MegAlign using CLUSTAL method algorithm in the DNASTAR package (Biocompare, Inc., CA, USA) and displayed in Genedoc [33] . The amino acid sequence of the *V. carchariae *chitinase was aligned with five selected bacterial chitinase sequences available in the Swiss-Prot or TrEMBL database (see Results). The secondary structure elements of *V. carchariae *chitinase A were obtained by the PHD method available in PredicProtein [34]. The modeled tertiary structure of the *Vibrio *chitnase was built by Swiss-Model and displayed by Swiss-Pdb Viewer [35] using the x-ray structure of *S. marcescens *chitinase A E315L mutant complex with hexaNAG (PDB code: 1NH6) as structure template. The co-ordinates of [GlcNAc]_6 _were modeled into the active site of the *Vibrio *enzyme and the target residues were located by superimposing the C_α _atoms of 459 residues of *V. carchariae *chitinase A with the equivalent residues of *S. marcescens *E315L complex, using the program Superpose available in the CCP4 suit [36]. The predicted structure was viewed with Pymol [37].

### Mutation design and site-directed mutagenesis

Site-directed mutagenesis was carried out by PCR using QuickChange site-directed mutagenesis kit (Stratagene). The pQE60 plasmid harbouring chitinase A DNA lacking the residues 598–850 *C*-terminal fragment was used as DNA template [25]. The primers (Bio Service Unit, Thailand) used for the mutagenesis are summarized in Table 3. The success of newly-generated mutations was confirmed by automated DNA sequencing (BSU, Thailand). The programs used for nucleotide sequence analyses were obtained from the DNASTAR package (DNASTAR, Inc., Madison, USA).

**Table 3:** Primers used for mutagenesis

**Point mutation**	**Oligonucleotide sequence**
Ser33→Ala	Forward 5'-CGATATGTACGGTGCG^a^AATAACCTTCAATTTTC-3'
	Reverse 5'-GAAAATTGAAGGTTATTCGCACCGTACATATCG-3'
Ser33→Trp	Forward 5'-CGATATGTACGGTTGGAATAAC**CTG**^b^CAATTTTC-3'
	Reverse 5'-GAAAATTG**CAG**GTTATTCCAACCGTACATATCG-3'
Trp70→Ala	Forward 5'-GAAATTTAACCAGGCGAGTGGCACATCTG-3'
	Reverse 5'-CAGATGTGCCACTCGCCTGGTTAAATTTC-3'
Trp231→Ala	Forward 5'-GTTATCCAT**GAT**CCGGCGgcagcttatc-3'
	Reverse 5'-GATAAGCTGCCGCCGG**ATC**ATGGATAAC-3'
Trp231→Phe	Forward 5'-GGTTATCCATGACCCGTTTGCAGCTTATCAG-3'
	Reverse 5'-CTGATAAGCTGCAAACGGGTCATGGATAACC-3'
Tyr245→Trp	Forward 5'-CAGGTCATGAATGGAGCACGCCAATCAAG-3'
	Reverse 5'-CTTGATTGGCGTGCTCCATTCATGACCTG-3'

^a ^Sequences underlined indicate the mutated codons.

^b ^Sequences in bold represent the codon being modified to achieve the *T*_m _value as required for QuickChange site-directed mutagenesis.

### Expression and purification of recombinant wild-type and mutant chitinases

The pQE60 expression vector harboring the DNA fragment that encodes wild-type chitinase A were highly expressed in *E. coli *M15 cells and the recombinant proteins purified as described elsewhere [28]. Briefly, the cells were grown at 37°C in Luria Bertani (LB) medium containing 100 μg/ml ampicillin until OD_600 _reached 0.6, and then 0.5 mM of isopropyl thio-*β*-D-galactoside (IPTG) was added to the cell culture for chitinase production. After 18 h of induction at 25°C, the cell pellet was collected by centrifugation, re-suspended in 15 ml of lysis buffer (20 mM Tris-HCl buffer, pH 8.0, containing 150 mM NaCl, 1 mM phenylmethylsulphonyl fluoride (PMSF), and 1.0 mg/ml lysozyme), and then lysed on ice using an Ultrasonic homogenizer. The supernatant obtained after centrifugation at 12,000 *g *for 1 h was instantly subjected to Ni-NTA agarose affinity chromatography following the Qiagen's protocol. After SDS-PAGE analysis [38], the chitinase containing fractions were pooled and then applied to Vivaspin-20 membrane filtration (*M*r 10 000 cut-off, Vivascience AG, Hannover, Germany) to concentrate the protein and to remove imidazole. A final concentration of the protein was determined by Bradford's method [39] using a standard calibration curve constructed from BSA (0–25 μg).

### Chitinase activity assays

The colorimetric assay was carried out in a 96-well microtiter plate using *p*NP-[GlcNAc]_2 _(Bioactive Co., Ltd., Bangkok, Thailand) as substrate. A 100-μl assay mixture, comprising protein sample (10 μl), 500 μM *p*NP-(GlcNAc)_2_, and 100 mM sodium acetate buffer, pH 5.5, was incubated at 37°C for 10 min with shaking. After the reaction was terminated by the addition of 1.0 M Na_2_CO_3 _(50 μl), the amount of *p*-nitrophenol (*p*NP) released was determined by A_405 _in a microtiter plate reader (Applied Biosystems, Foster City, CA, USA). Molar concentrations of the *p*NP product were estimated from a calibration curve of *p*NP (0–30 nmol). Alternatively, chitinase activity was measured by a reducing-sugar assay. The reaction mixture (500 μl), containing 1% (w/v) colloidal chitin (prepared based on Hsu & Lockwood [40]), 100 mM sodium acetate buffer, pH 5.5, and 100 μg chitinase A, was incubated at 37°C in a Thermomixer comfort (Eppendorf AG, Hamburg, Germany). After 15 min of incubation, the reaction was terminated by boiling at 100°C for 5 min, and then centrifuged at 5,000 g for 10 min to precipitate the remaining chitin. A 200-μl supernatant was then subjected to DMAB assay following Bruce *et al*. [41]. The release of the reducing sugars as detected by A_585 _was converted to molar quantity using a standard calibration curve of [GlcNAc]_2 _(0–1.75 μmol). For crystalline *α*-chitin, chitinase activity assay was carried out as described for colloidal chitin with 400 μg of chitinase A included in the assayed mixture.

### Chitin binding assays

Chitin binding assays were carried out at 0°C to minimize hydrolysis. For time course studies, a reaction mixture (500 μl), containing 1.0 μmol enzyme, and 1.0 mg of chitin in 20 mM Tris-HCl buffer, pH 8.0, was incubated to a required time of 0, 1.25, 2.5, 5, 10, 15, 20, 25, and 30 min, and then the supernatant was collected by centrifuging at 12000 g at 4°C for 10 min. Concentration of the remaining enzyme was determined by Bradford's method, while concentration of the bound enzyme (E_b_) was calculated from the difference between the initial protein concentration (E_t_) and the free protein concentration (E_f_) after binding.

The chitin binding assay was also carried out with crystalline chitin and colloidal chitin (Sigma-Aldrich Pte Ltd., The Capricorn, Singapore Science Park II, Singapore) as tested polysaccharides. A reaction (set as above) was incubated for 60 min at 0°C, then the chitin-bound enzyme was removed by centrifugation, and the concentration of the free enzyme was determined. For adsorption isotherm experiments, the reaction assay (also prepared as described above) containing varied concentrations of protein from 0 to 7.0 μM was incubated for 60 min. After centrifugation, concentration of free enzyme in the supernatant was determined. A plot of [E_b_] vs [E_f_] was subsequently constructed and the dissociation binding constants (*K*_d_) of wild-type and mutants were estimated using a non-linear regression function in the GraphPad Prism software (GraphPad Software Inc., San Diego, CA).

### Steady-state kinetics

Kinetic parameters of the chitinase variants were determined using chitohexaose or colloidal chitin as substrate. For chitohexaose, the reaction mixture (200 μl), containing 0–500 μM (GlcNAc)_6_, and 50 μg enzyme in 100 mM sodium acetate buffer, pH 5.5, was incubated at 37°C for 10 min. After boiling to 100°C for 3 min, the entire reaction mixture was subjected to DMAB assay as described earlier. For colloidal chitin, the reaction was carried out the same way as the reducing-sugar assay, but concentrations of colloidal chitin were varied from 0 to 5.0% (w/v). The amounts of the reaction products produced from both substrates were determined from a standard curve of [GlcNAc]_2 _(0–1.75 μmol). The kinetic values were evaluated from three independent sets of data using the nonlinear regression function obtained from the GraphPad Prism software.

## Abbreviations

GlcNAc_n_: *β*-1–4 linked oligomers of *N*-acetylglucosamine residues where n = 1–6; DMAB: *p*-dimethylaminobenzaldehyde; IPTG: Isopropyl thio-*β*-D-galactoside; PMSF: Phenylmethylsulphonylfluoride.

## Authors' contributions

SP performed site-directed mutagenesis, recombinant expression, protein purification, and functional characterization. CS carried out the sturcture-based sequence alignment and the molecular modeling of the tertiary structure of V. carchariae chitinase A. WS initiated the ideas of research, was involved in primer design and site-directed mutagenesis, performed data analyses, and prepared the manuscript.
